# Kinsenoside Alleviates 17α-Ethinylestradiol-Induced Cholestatic Liver Injury in Rats by Inhibiting Inflammatory Responses and Regulating FXR-Mediated Bile Acid Homeostasis

**DOI:** 10.3390/ph14050452

**Published:** 2021-05-11

**Authors:** Jiaxiong Ming, Qianqian Xu, Limin Gao, Yanfang Deng, Jie Yin, Qun Zhou, Qingyi Tong, Yonghui Zhang

**Affiliations:** 1 Hubei Key Laboratory of Natural Medicinal Chemistry and Resource Evaluation, School of Pharmacy, Tongji Medical College, Huazhong University of Science and Technology, Wuhan 430030, China; jxminghust@163.com (J.M.); xuqq@hust.edu.cn (Q.X.); D201981424@hust.edu.cn (Y.D.); D202081574@hust.edu.cn (J.Y.); 2 Biobank, Union Hospital, Tongji Medical College, Huazhong University of Science and Technology, Wuhan 430022, China; gaolimin@hust.edu.cn

**Keywords:** kinsenoside, 17α-ethinylestradiol, inflammation, cholestasis

## Abstract

Cholestasis is an important predisposing factor of liver diseases, such as hepatocyte necrosis, liver fibrosis and primary biliary cirrhosis. In this study, we aimed to investigate the effects of Kinsenoside (KD), a natural active ingredient of *Anoectochilus roxburghii*, on estrogen-induced cholestatic liver injury in Sprague-Dawley rats and the underlying mechanism. The rats were randomly divided into six groups: control group, model group, low-dose KD group (50 mg/kg body weight, KD-L), medium-dose KD group (100 mg/kg body weight, KD-M), high-dose KD group (200 mg/kg body weight, KD-H) and ursodeoxycholic acid group (40 mg/kg body weight, UDCA). 17α-Ethinylestradiol (EE) was used to establish an experimental animal model of estrogen-induced cholestasis (EIC). The results demonstrated that KD alleviated liver pathologic damage, serum biochemical status and inhibited hepatocellular microstructure disorder and bile duct hyperplasia in EE-induced cholestatic rats. Mechanically, KD alleviated EE-induced cholestatic liver injury by inhibiting inflammatory responses and regulating bile acid homeostasis. Concretely, KD reduced the expression of IL-1β and IL-6 by inhibiting NF-κB p65 to suppress EE-mediated inflammation in rat liver. KD enhanced the expression of FXR and inhibited EE-mediated reduction of FXR in vitro and in vivo. It was the potential mechanism that KD mitigates cholestasis by increasing efflux and inhibiting uptake of bile acids via FXR-mediated induction of bile salt export pump (BSEP) and reduction of Na^+^-dependent taurocholate cotransport peptide (NTCP) to maintain bile acid homeostasis. Moreover, KD repressed the bile acid synthesis through reducing the expression of synthetic enzyme (CYP7A1), thereby normalizing the expression of metabolic enzyme (SULT2A1) of bile acid. In conclusion, our results revealed that KD may be an effective drug candidate for the treatment of cholestasis.

## 1. Introduction

Cholestasis is a clinically pathological condition characterized by excessive accumulation of toxic bile acids (BAs) in liver due to various causes, including viral hepatitis [1], autoimmune liver disease [2], alcoholic and/or fatty liver disease [3,4], as well as the induction of chemicals, such as estrogen [5,6]. Estrogen-induced cholestasis (EIC) is common in women undergoing oral contraceptives, pregnancy or hormone replacement therapy [7,8,9] and even in men receiving estrogen therapy for prostate cancer [10]. Cholestasis can induce liver cell necrosis, liver fibrosis, primary biliary cirrhosis (PBC) and even liver failure [11,12,13]. Currently, there are very few effective drugs and therapies for cholestasis. Ursodeoxycholic acid (UDCA) is the first drug approved by the FDA for the treatment of PBC. UDCA can effectively treat more than 50% of PBC patients; however, more than 40% of patients did not respond well and 5% to 10% of patients were intolerant [14]. Obeticholic acid (OCA), the second medicament, was approved by FDA in 2016 to treat patients of PBC, who are intolerant or had an inadequate response to UDCA [15]. Therefore, it is of great significance to develop new drugs and strategies for the clinical treatment of cholestatic liver injury.

17α-ethinylestradiol (EE), a synthetic estrogen, is widely used in rats to establish experimental intrahepatic cholestasis model and to study the molecular mechanism involved in EIC. EE leads to cholestatic liver injury by activating estrogen receptor α to cause the suppression of bile acid transporters and the alteration of bile acid synthetic enzymes [16]. It has been demonstrated that EE suppresses biliary secretion via reducing the expression of bile salt export pump (BSEP) and Na^+^-dependent taurocholate cotransporter (NTCP) and increased accumulation of toxic bile acids by altering synthetic pathway of cytochrome P450 7A1 (CYP7A1) [17]. On the other hand, EE also leads to an inflammatory reaction accompanied with increasing expression of IL-1β and IL-6 in the liver [18]. Therefore, it is of great importance for the drug candidate to exert anti-inflammatory pharmacological effects on estrogen-induced cholestasis. The homeostasis of bile acids is highly regulated by nuclear receptors including farnesoid X receptor (FXR), pregnane X receptor (PXR), vitamin D receptor (VDR) and constitutive androstane receptor (CAR) [19]. Among them, FXR is a member of the superfamily of transcription factors activated by ligands that are highly expressed in the liver and intestine, and plays a vital role in maintaining the bile acid homeostasis [20]. FXR induces the expression of BSEP to enhance the efflux of bile acids and inhibits the expression of NTCP to reduce hepatic uptake of bile acids [21]. In addition, FXR suppresses CYP7A1 to decrease bile acid synthesis and induces SULT2A1 to accelerate detoxification of bile acids in liver [22,23]. Studies have also indicated that EE reduces the expression of FXR in vivo and in vitro [24]. Hence, it is an important therapeutic strategy for the treatment of cholestasis to reverse the EE-mediated reduction in FXR.

**Figure 1:**
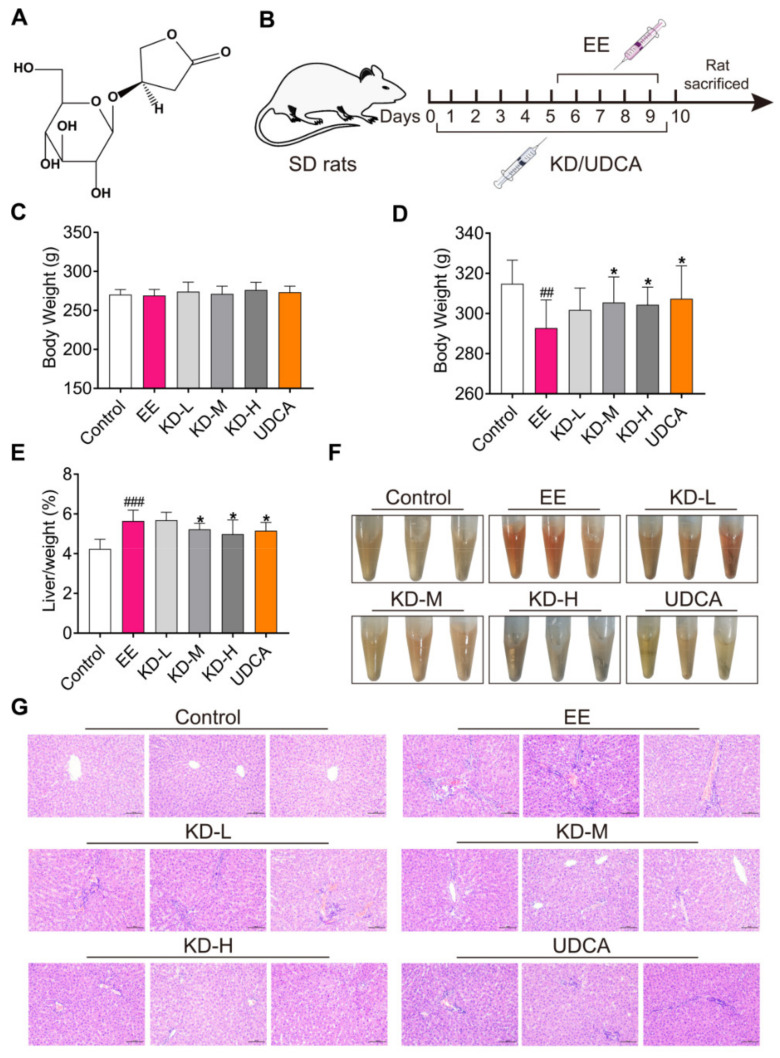
The therapeutic effect of KD on EE-induced cholestatic liver injury in rats. (**A**) The chemical structure of Kinsenoside (KD). (**B**) Schematic of the experiment. (**C**) The body weight of rats in each group before KD or UDCA treatment. (**D**) Changes in body weight of rats in each group at the end of KD or UDCA administration. (**E**) The ratio of liver weight to body weight in each group was measured when rats were sacrificed. (**F**) Representative images of phenotype of serum from each group were exhibited after receiving KD-L, KD-M, KD-H and UDCA treatment, respectively. (**G**) Pathological images of liver in each group were analysed by hematoxylin and eosin (H&E) staining. Scale bar, 100 μm. In (**C**–**E**), data are presented as mean ± SD (*n* = 10). ^##^ *p* < 0.01 and ^###^ *p* < 0.001 denote statistical significance, compared to the control group. * *p* < 0.05 denotes statistical significance, compared to the EE group.

*Anoectochilus roxburghii*, a perennial herb of the Orchidaceae, has been used as a natural medicinal plant to treat a variety of diseases, including type 2 diabetes mellitus, chronic hepatitis B, hyperuricemia and hand-foot-and-mouth disease [25]. It has been demonstrated that Kinsenoside (KD, the chemical structure was exhibited in Figure 1A) extracted from *Anoectochilus roxburghii,* is a small molecule active ingredient that has multiple pharmacological effects on anti-hyperlipidemia [26], anti-inflammatory [27], vascular protection [28] and hepatoprotective activity [29,30]. In the present study, we aimed to investigate the effect of KD against EIC, and further understand the potential mechanisms in vivo and in vitro. KD inhibits inflammatory responses and regulates bile acid homeostasis to alleviate EE-induced cholestatic liver injury in rats by reducing the expression of IL-1β, IL-6 and suppressing EE-mediated reduction in FXR in rats. The findings of our study suggested that KD might be a novel and potentially effective drug candidate for the treatment of cholestasis.

## 2. Results

### 2.1. KD Protects against EE-Induced Cholestatic Liver Injury in Rats

In order to investigate the effect of KD on cholestatic liver injury in vivo, we established an experimental animal model of estrogen-induced cholestasis (Figure 1B). Rats were subcutaneously injected with EE (10 mg/kg body weight) once daily for five consecutive days, as previously described [31]. The low-dose KD (50 mg/kg body weight, KD-L), medium-dose KD (100 mg/kg body weight, KD-M), high-dose KD (200 mg/kg body weight, KD-H) and ursodeoxycholic acid (40 mg/kg body weight, UDCA) were orally gavaged in relevant group. The body weight of rats in all groups was 250–300 g before administration, and there was no significant difference in body weight in each group (Figure 1C). Compared with the control group, the EE group experienced a significant reduction in body weight after continuous subcutaneous injection of estrogen. However, compared with the EE group, the medium and high doses of KD and UDCA, a positive treatment drug, could significantly inhibit the weight loss induced by EE (Figure 1D). In addition, compared with the control group, EE obviously increased the liver body index of rats, while medium and high doses of KD and UDCA not only evidently improved the liver body index, but also normalized the serum color (Figure 1E,F). Moreover, histopathological analysis of rat livers found that EE visibly increased inflammatory cell infiltration, bile duct proliferation, oedema and hepatocyte necrosis. KD relieved the liver pathological symptoms induced by EE in a dose-dependent manner, and UDCA also effectively improved the occurrence of liver pathology (Figure 1G). These results showed that KD protects from EE-induced cholestatic liver injury in rats.

### 2.2. KD Normalizes Cholestasis-Related Serum Biochemical Factors

**Figure 2:**
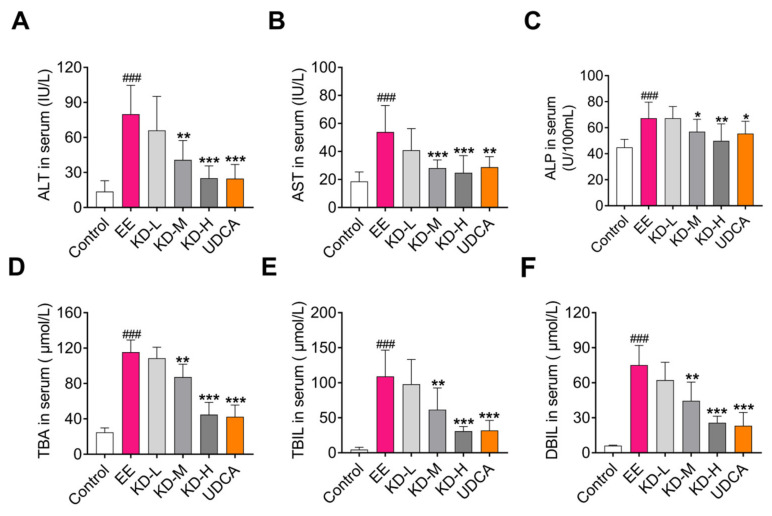
The effects of KD on serum biochemical factors associated with EE-induced cholestasis in rats. Serum (**A**) alanine aminotransferase (ALT), (**B**) aspartate aminotransferase (AST), (**C**) alkaline phosphatase (ALP), (**D**) total bile acids (TBA), (**E**) total bilirubin (TBIL) and (**F**) direct bilirubin (DBIL) levels in each group were determined by commercial kits. Data are presented as mean ± SD (*n* = 10). ^###^ *p* < 0.001 denotes statistical significance, compared to the control group. * *p* < 0.05, ** *p* < 0.01 and *** *p* < 0.001 denote statistical significance, compared to the EE group.

Alanine aminotransferase (ALT) and aspartate aminotransferase (AST) are considered monitoring indicators of liver function impairment caused for various reasons. Serum levels of total bile acids (TBA), bilirubin (BIL) and alkaline phosphatase (ALP) are biochemical indicators of cholestatic liver injury. Here, we tested these cholestasis-related serum biochemical factors using the commercial kits. The results showed that EE significantly increased the content of preceding serum factors compared with the control group, which also indicated that EE has successfully induced cholestasis in rats. However, these indicators were dose-dependently reduced by KD treatment (Figure 2A–F). UDCA, as a positive therapeutic drug, also inhibited the increase in serum biochemical factors induced by EE. Taken together, these results suggested that KD has protective effects on EE-induced liver injury in rats.

### 2.3. KD Inhibits Hepatocellular Microstructural Disorder and Bile Duct Cell Proliferation

**Figure 3:**
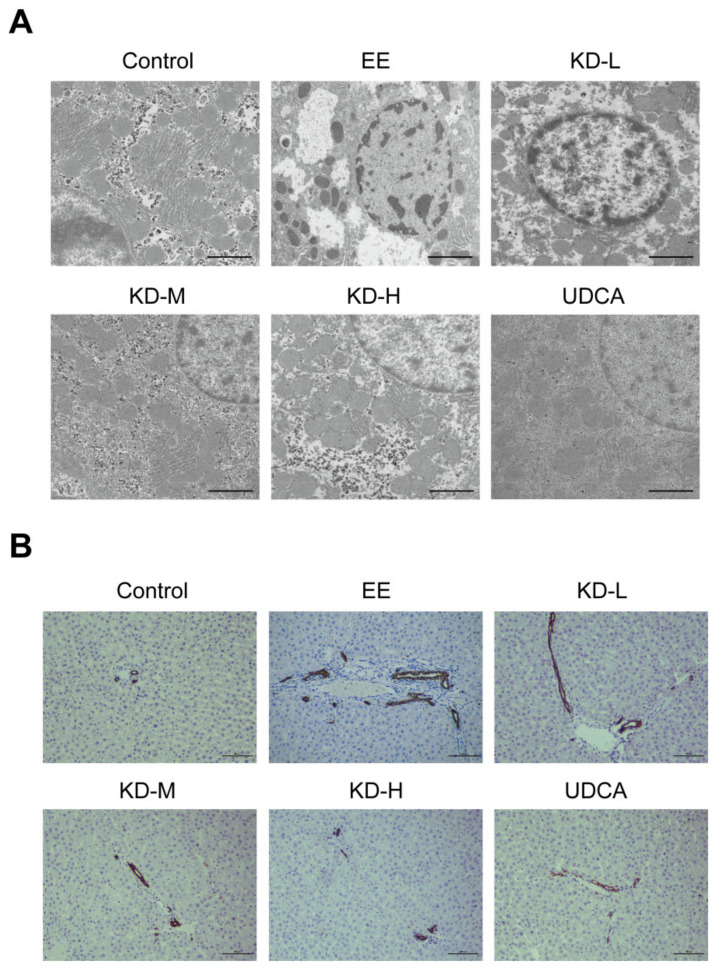
The effects of KD on hepatocellular microstructure and bile duct cell proliferation. (**A**) Representative images of liver in each group by TEM were analysed to assess the microstructural changes in hepatocytes. Scale bar, 2 μm. (**B**) Representative immunohistochemical images of CK19 expressed in bile duct cells from each group were acquired to evaluate hyperplasia of the bile duct. Scale bar, 100 μm.

To definitely study the damages of EE in hepatocytes, we performed transmission electron microscopy (TEM) on liver tissue to observe the microstructural changes in hepatocytes. Compared with the control group, it was shown in hepatocyte that EE significantly reduced the distribution of perinuclear endoplasmic reticulum and mitochondria and resulted in a severe deficiency of cytoplasm. However, these changes were reversed by treatment with KD. It was KD that not only regained the number and distribution of perinuclear cell organelles, but also improved the morphology in hepatocytes (Figure 3A). CK19 is a cytokeratin that is specifically expressed in bile duct and is a biomarker of bile duct cells [32]. In EE-treated rats, we found extensive expression of CK19, which was an indicator of bile duct cell proliferation induced by cholestasis; however, these pathological changes were evidently reduced by KD or UDCA treatment (Figure 3B).

### 2.4. KD Reduces the Expression of Pro-Inflammatory Cytokines Caused by EE in the Liver

**Figure 4:**
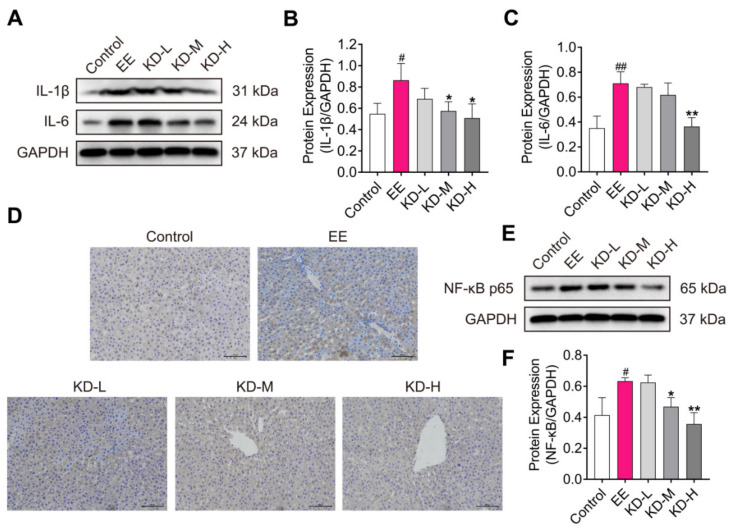
The effect of KD on inflammatory reaction in the liver. (**A**) The protein expression of IL-1β and IL-6 in rat liver was detected using Western blotting. (**B**,**C**) The relative protein expression of IL-1β and IL-6 were analysed by using Image J software. (**D**) Immunohistochemistry staining of NF-κB p65 in the liver. Brown color indicates NF-κB p65-activated cells and blue color indicates cell nuclei. Scale bar, 100 μm. (**E**) The protein expression of NF-κB p65 in the liver was detected by using western blotting. (**F**) The relative protein expression of NF-κB p65 was analysed by using Image J software. Data are presented as mean ± SD (*n* = 3). ^#^ *p* < 0.05 and ^##^ *p* < 0.01 denote statistical significance, compared to the control group. * *p* < 0.05 and ** *p* < 0.01 denote statistical significance, compared to the EE group.

Cholestasis is also an inflammatory disease characterized by elevated IL-1β and IL-6 levels in liver [33]. IL-1β and IL-6 are pro-inflammatory cytokines that play roles in EE-induced cholestatic liver injury. The protein expression of IL-1β and IL-6 was significantly increased in the EE-treated group compared to the control group, however, which were distinctly dose-dependently decreased in KD-treated groups (Figure 4A–C). NF-κB p65 is a key transcription factor that regulates many inflammatory cytokines, including IL-1β and IL-6, to mediate the inflammatory processes [34]. Immunohistochemistry of NF-κB p65 demonstrated that there was a higher positive expression level in EE-treated group compared with the control group, but KD evidently reversed this effect in a dose-dependent manner (Figure 4D). Consistent with immunohistochemistry, the protein expression of NF-κB p65 was also obviously decreased by KD treatment (Figure 4E,F). These results suggested that KD alleviates EE-induced cholestatic liver injury by inhibiting inflammatory responses in rats.

### 2.5. KD Suppresses EE-Mediated Decrease in FXR In Vitro and In Vivo

**Figure 5:**
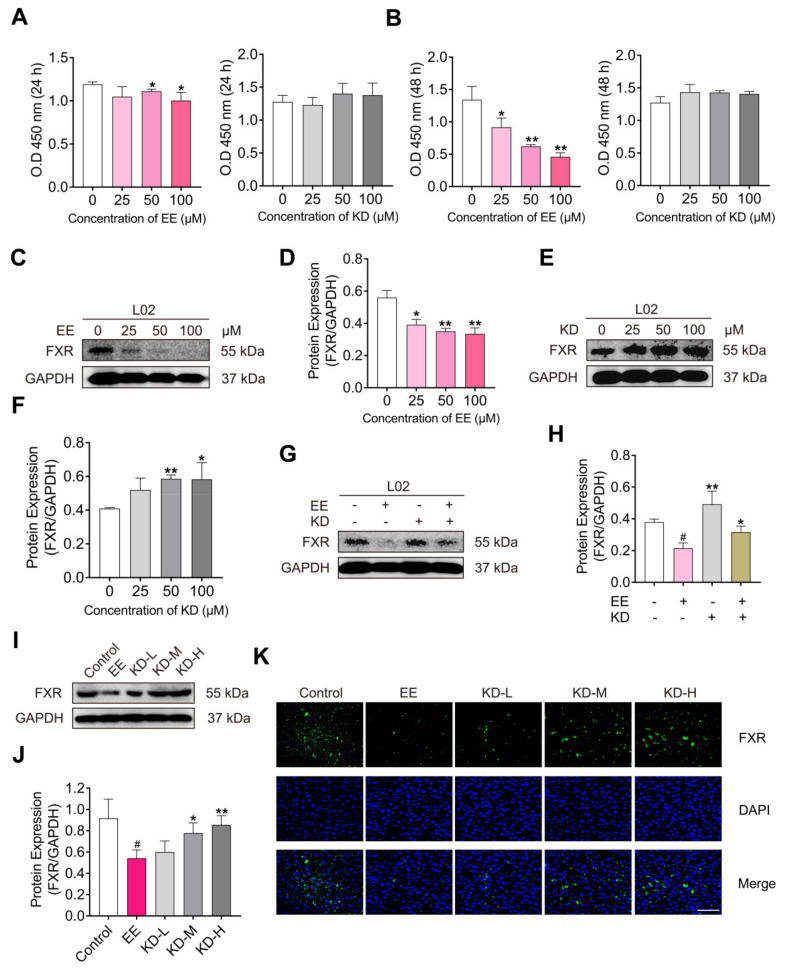
The effects of KD on the protein expression of FXR in human hepatocyte cell line L02 cells and rat liver with EE-induced cholestasis. (**A**,**B**) L02 cells were treated with EE or KD at different concentrations (0, 25, 50, 100 μM) for 24 or 48 h. The values of O.D 450 nm were measured by CCK-8 assay to indicate cell viability. (**C**) The protein expression of FXR in L02 cells treated with EE (0, 25, 50, 100 μM) for 48 h was detected using Western blotting. (**D**) The relative protein expression of FXR was analysed by using Image J software. (**E**) The protein expression of FXR in L02 cells treated with KD (0, 25, 50, 100 μM) for 48 h was detected using Western blotting. (**F**) The relative protein expression of FXR was analysed by using Image J software. (**G**) The protein expression of FXR in L02 cells treated with EE (25 μM, +), KD (25 μM, +) or EE combined with KD for 48 h was detected using Western blotting. (**H**) The relative protein expression of FXR was analysed by using Image J software. (**I**) The protein expression of FXR in rat liver was detected using Western blotting. (**J**) The relative protein expression of FXR was analysed by using Image J software. (**K**) Immunofluorescence staining of FXR in the liver. Green color indicates FXR and blue color indicates cell nuclei. Scale bar, 100 μm. Data are presented as mean ± SD (*n* = 3). In (**A**,**B**,**D**,**F**), * *p* < 0.05 and ** *p* < 0.01 denote statistical significance, compared to the control group. In (**H**,**J**), ^#^ *p* < 0.05 denotes statistical significance, compared to the control group. * *p* < 0.05 and ** *p* < 0.01 denote statistical significance, compared to the EE group.

FXR, a transcription factor highly expressed in the liver and intestine, is considered to be one of the most important regulatory factors in maintaining bile acid homeostasis. In the present study, CCK-8 assay and Western blotting were performed to investigate the effects of EE and KD on the cell viability and the expression of FXR in vitro. The normal human hepatocyte cell line L02 cells were treated with a gradient concentration of EE, KD for 24, 48 h. The results revealed that EE significantly attenuated the viability of L02 cells as the concentration increases. However, the cell viability was not subjected to the gradient concentration of KD (Figure 5A,B). The data elucidated that KD possesses property of low cytotoxicity. Subsequently, we assessed the expression of FXR in L02 cells exposed to different doses of EE, KD for 48 h. The Western blotting results demonstrated that EE significantly reduced the expression of FXR in a dose-dependent manner (Figure 5C,D). In contrast, KD obviously promoted the expression of FXR with increasing concentration (Figure 5E,F). Additionally, to research the effects of KD treatment on EE-mediated decrease in FXR, L02 cells were treated with EE, KD or EE combined with KD for 48 h. The results suggested that EE, combined with KD, evidently reversed the decrease in FXR due to EE treatment (Figure 5G,H). Therefore, these results potentially indicated that KD suppresses the EE-mediated decrease in FXR in vitro and in vivo. The Western blotting assay revealed that KD suppressed the EE-mediated reduction in FXR in the liver (Figure 5I,J). Moreover, there were consistent results in the immunofluorescence staining assay, showing that FXR was obviously inhibited in EE-treated group compared with the control group. However, FXR was increased in KD-treated groups compared with the EE group (Figure 5K).

### 2.6. KD Alters the Expression of Transporters, Synthetic and Metabolic Enzyme Involved in Bile Acid Homeostasis

Bile acid homeostasis is regulated by the hepatobiliary transporters, bile acid synthetic and metabolic enzymes.

**Figure 6:**
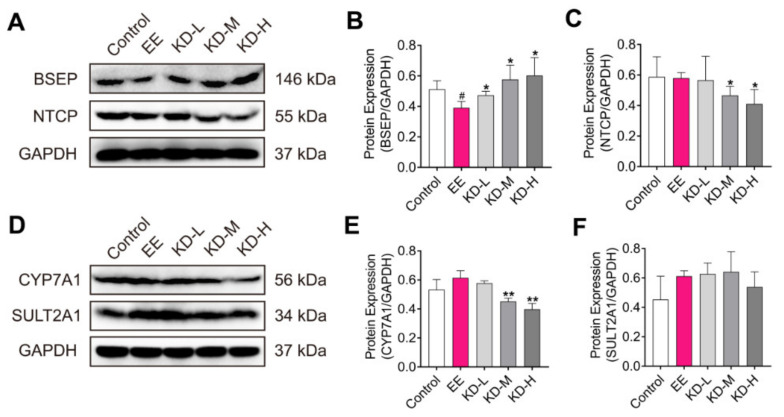
The effects of KD on the expression of the factors regulating transport, synthesis and metabolism of bile acids in the liver with EE-induced cholestasis. (**A**) The protein expression of BSEP and NTCP in rat liver was detected using Western blotting. (**B**,**C**) The relative protein expression of BSEP and NTCP were analysed using Image J software. (**D**) The protein expression of CYP7A1 and SULT2A1 in rat liver was detected using Western blotting. (**E**,**F**) The relative protein expression of CYP7A1 and SULT2A1 were analysed using Image J software. Data are presented as mean ± SD (*n* = 3). ^#^ *p* < 0.05 denotes statistical significance, compared to the control group. * *p* < 0.05 and ** *p* < 0.01 denote statistical significance, compared to the EE group.

Bile salt export pump (BSEP) and Na^+^-dependent taurocholate cotransport peptide (NTCP) are important transporters located in the membrane of hepatocyte. BSEP mediates the transport of bile acids across the hepatocyte canalicular membrane and regulates bile acid-dependent bile secretion [35]. NTCP plays a role in the hepatic sodium/bile acid uptake system, which functions as a substrate-specific, sodium-dependent transporter of both bile and non-bile organic compounds [36]. Cytochrome P450 7A1 (CYP7A1), a bile acid synthetic enzyme, and sulfotransferase 2A1 (SULT2A1), a phase II enzymes of bile acid detoxification, play important roles in the bile acid homeostasis involved in synthesis and metabolism of bile acids, respectively [37]. To further elucidate the mechanism of the amelioration of EE-induced cholestasis by KD, the expression levels of BSEP, NTCP, CYP7A1 and SULT2A1 in rat liver were determined by Western blotting. Our results revealed that KD treatment increased the expression of BSEP and restrained the expression of NTCP and CYP7A1 in a dose-dependent manner compared to the EE group (Figure 6A–E). Meanwhile, we found that the expression of SULT2A1 was enhanced by EE treatment, which may result from the increase in the compensatory metabolism of bile acid in the liver due to cholestasis. As the dose of KD increased, the expression of SULT2A1 tended towards the normalized level instead of significantly increasing to enhance the metabolism of bile acids (Figure 6F). This may be the result of KD treatmentmaintained bile acid homeostasis in hepatocyte by increasing efflux, reducing intrahepatic intake and the synthesis of bile acids. Combining the previous results, the potential mechanism is that KD maintains bile acid homeostasis by increasing efflux, and inhibiting the uptake and synthesis of bile acids via the FXR-mediated induction of BSEP, reduction in NTCP and suppression of CYP7A1, thereby alleviating EE-induced cholestasis and normalizing the expression of metabolic enzyme (SULT2A1) of bile acid in the liver.

## 3. Discussion

Cholestatic liver diseases, including estrogen-induced cholestasis (EIC), primary biliary cirrhosis (PBC) and primary sclerosing cholangitis (PSC), are syndromes associated with intrahepatic retention and the accumulation of bile acids (BAs) [38]. If there is no timely treatment, long-term cholestasis will cause liver fibrosis, cirrhosis and even liver failure [39]. EIC is common in pregnant women, and there is generally a 0.2–2% chance that they suffer from intrahepatic cholestasis of pregnancy [8]. Currently, only ursodeoxycholic acid (UDCA) and obeticholic acid (OCA) were approved by the Food and Drug Administration (FDA, USA) as therapeutic medicine for patients diagnosed with cholestasis [40]. It has been demonstrated that UDCA, in the treatment of cholestasis, may result from a combination of anti-cholestatic, cytoprotective, anti-apoptotic, and immunodulatory effects [41]. However, up to 40% of patients of PBC have an inadequate response to UDCA therapy [42]. OCA has been evaluated as a second-line therapy in PBC patients who have an inadequate or intolerant response to UDCA [15]. Hence, developing a new drug candidate for the treatment of cholestasis is of necessity. Recently, increasing studies have demonstrated that natural products are a valuable resource when seeking novel therapeutic agents to prevent and treat cholestatic liver diseases [43]. Herein, kinsenoside (KD) is a natural active compound extracted from *Anoectochilus roxburghii*, a renowned medicinal plant. In this work, we demonstrated the protective effect of KD on EE-induced cholestatic liver injury in rats based on previous studies showing that KD possesses observable anti-inflammatory [44] and hepatoprotective activity [45]. Our study revealed that KD not only ameliorated liver histopathological damage, liver body index and serum biochemistry, but also inhibited hepatocellular microstructural disorder and bile duct cell proliferation in the experimental animal model of EE-induced cholestasis. Therefore, our data indicated that KD may be a potential drug candidate for the treatment of cholestasis.

Inflammation usually occurs in EIC, and increased inflammatory cytokines accelerate the development and progression of cholestasis [46]. It has been reported that KD alleviates acute inflammatory hazards by inhibiting the production of inflammatory mediators, and enhanced anti-inflammatory cytokine generation in vitro and in vivo [47]. Previous studies also revealed that KD attenuates gouty arthritis and osteoarthritis through inactivating the NF-κB signaling pathway [44,48]. To elucidate the pharmacological effects of KD on the alleviation of EE-induced cholestasis, we tested the protein expression of IL-1β and IL-6, known as pro-inflammatory cytokines, by Western blotting. Our data showed that IL-1β and IL-6 were prominently reduced in liver with EE-induced cholestasis by KD treatment. The effects of KD on inflammatory responses may result from the suppression of NF-κB, a key transcription factor that promotes the production of inflammatory cytokines. In addition, it also revealed that KD ameliorates intervertebral disc degeneration through the activation of AKT-ERK1/2-Nrf2 signaling pathway [49]. Moreover, there was also evidence indicating that KD suppresses AGEs-induced endothelial dysfunction via the AGEs/RAGE/NF-κB pathway and oxidative stress-induced cell apoptosis via Erk/p38/NF-κB signaling [50,51]. Increasing evidence has demonstrated that KD, a promising molecule, may be considered as drug candidate to treat a variety of diseases, including liver diseases [52].

**Figure 7:**
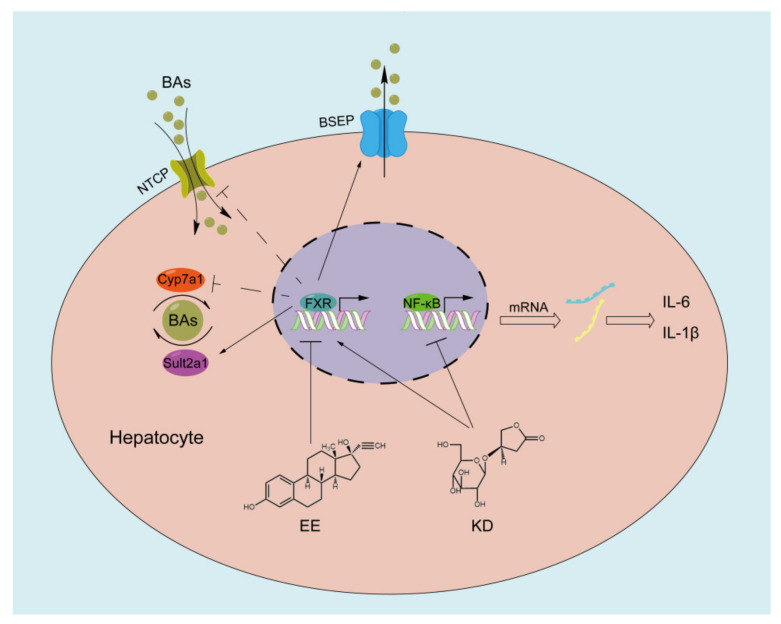
Schematic diagram of potential working mechanisms that KD alleviates EE-induced cholestatic liver injury.

The development and progression of cholestasis often means a dysfunction of bile acid homeostasis. Farnesoid X receptor (FXR) is one of the most important regulators for maintaining bile acid homeostasis, manifesting in transport, synthesis and metabolism of bile acid [53]. FXR transcriptional activation directly induces the expression of the bile salt export pump (BSEP), which is located at the canalicular membrane of hepatocytes and promotes bile acid transport from hepatocytes into bile. FXR represses the expression of Na^+^-dependent taurocholate cotransport peptide (NTCP) to reduce uptake of bile acid from portal blood into hepatocytes and the expression of cytochrome P450 7A1 (CYP7A1) to decrease bile acid synthesis in hepatocyte by activating the hepatic small heterodimer partner (SHP), a direct target gene of FXR. Meanwhile, FXR promotes sulfotransferase 2A1 (SULT2A1), a phase II enzymes, expression to increase bile acid metabolism. Therefore, targeting FXR is considered a promising treatment strategy for cholestasis [54]. In our study, the results demonstrated that 17α-ethinylestradiol (EE) observably suppressed the expression of FXR in vitro and in vivo, which was responsible for the development of cholestasis. However, we found that KD enhanced the expression of FXR and inhibited EE-mediated reduction in FXR in vitro and in vivo. Based on the results, here, our research provides an innovative understanding of KD in the aspect of liver protection and attempts to reveal the possible mechanism of the alleviation of EE-induced cholestatic liver injury through KD treatment. EE disturbs bile acid homeostasis in hepatocytes by inhibiting the expression of FXR, thereby promoting the development and progression of cholestasis. The accumulation of toxic bile acids in hepatocytes accelerates NF-κB-mediated liver inflammation by upregulating pro-inflammatory cytokines including IL-1β and IL-6. In contrast, KD promotes the expression of FXR in hepatocytes to maintain bile acid homeostasis through the induction of BSEP and reduction in NTCP, CYP7A1, in spite of SULT2A1 at a normalized level, and reduces the expression of IL-1β and IL-6 in hepatocytes by suppressing the NF-κB-mediated inflammatory responses (Figure 7).

In summary, although KD exerts therapeutic benefits at higher doses, our unpublished results also demonstrated that KD has significant antioxidant activity and low toxicity in vitro and in vivo. Judging from all the findings, KD is approximately equal activity to UDCA for the treatment of EE-induced cholestatic liver injury. Our work suggested that KD, an active small molecule extracted from *Anoectochilus roxburghii,* alleviates 17α-ethinylestradiol-induced cholestatic liver injury in rats by inhibiting inflammatory responses and regulating FXR-mediated bile acid homeostasis. KD should be furtherly investigated as a promising drug candidate for the treatment of cholestatic liver diseases.

## 4. Materials and Methods

### 4.1. Chemicals and Reagents

Kinsenoside (KD, purity > 98%) was isolated from intact *Anoectochilus roxburghii* from Fujian Province of China. After being crushed, the *Anoectochilus roxburghii* was extracted with methanol under heating and refluxing. The methanol extracts were filtered and concentrated in a rotary evaporator. An appropriate amount of water was added to the methanol extracts, which were sequentially extracted with petroleum ether, ethyl acetate and n-butanol. The n-butanol extracts and the water extracts were, respectively, subjected to gradient elution with a chloroform-methanol system and adsorption resin column chromatography, and finally, the separating extracts were recrystallized to obtain KD. The purification of KD was determined by thin-layer chromatography (TLC), high-performance liquid chromatography (HPLC) and melting point and optical rotation determination. The data of structure of KD can be obtained from our previous studies [55]. 17α-Ethinylestradiol was purchased from Aladdin Biochemical Technology Co., Ltd. (Shanghai, China). Alanine aminotransferase (ALT), aspartate aminotransferase (AST), alkaline phosphatase (ALP), total bile acids (TBA), total bilirubin (TBIL) and direct bilirubin (DBIL) detection kits were obtained from Nanjing Jiancheng Institute of Biotechnology (Nanjing, China). Anti-FXR, anti-BSEP and anti-CYP7A1 antibodies were purchased from Santa Cruz Biotechnology (Dallas, TE, USA). Primary antibodies including NF-κB p65 and GAPDH were purchased from Cell Signaling Technology (Danvers, MA, USA), and primary antibodies against IL-1β, IL-6, NTCP and SULT2A1 were purchased from Absin Biotechnology Co., Ltd. (Shanghai, China).

### 4.2. Animals and Treatments

Male Sprague-Dawley rats weighing 220 ± 20 g were obtained from SPF Biotechnology Co., Ltd. (Beijing, China). All rats were adequately provided a standard laboratory diet and water in specific pathogen-free conditions under a temperature of 25 ± 2 °C with relative humidity at 60–70% and 12 h light/dark cycle in the Experimental Animal Center of Huazhong University of Science and Technology. Animal experimental procedures were approved (Permit Number: S2321) by the Institutional Animal Care and Use Committee of Tongji Medical College of Huazhong University of Science and Technology. After acclimatization for a week, the rats were randomly divided into six groups (*n* = 10). The control group and EE group were orally gavaged with equal volume of normal saline. Rats in KD group were orally gavaged with 50 mg/kg body weight (KD-L), 100 mg/kg body weight (KD-M) and 200 mg/kg body weight (KD-H) of KD, respectively. Rats in positive control group were orally gavaged with 40 mg/kg body weight of UDCA. All rats were administered once a day for a total of ten days. Since the sixth day, rats were injected subcutaneously with EE (10 mg/kg body weight) once daily for five consecutive days, excluding the control group, injected subcutaneously with vehicle (80% 1, 2-propanediol with 0.15% NaCl). After 24 h EE or vehicle administration, all rats were sacrificed, and the liver and blood were collected for further assays.

### 4.3. Histopathology Assay

After rats were sacrificed, liver samples were dissected, fixed in 4% paraformaldehyde and embedded in paraffin. Paraffin blocks of liver tissue were cut into 5 μm sections and stained with Hematoxylin and Eosin (H&E). Images to detect histopathological changes were taken using an optical microscope (ECLIPSE E100, Nikon, Japan).

### 4.4. Serum Biochemistry Assay

Briefly, blood samples of rats were collected into sterile tubes and were of immovability at room temperature for 2 h, and then serum, were separated from blood samples by centrifuging at 4 °C for 10 min, which levels of alanine aminotransferase (ALT), aspartate aminotransferase (AST), alkaline phosphatase (ALP), total bile acids (TBA), total bilirubin (TBIL) and direct bilirubin (DBIL) were detected using commercial kits purchased from Nanjing Jiancheng Institute of Biotechnology (Nanjing, China), according to the manufacturer’s protocols.

### 4.5. Transmission Electron Microscopy

Liver samples (2 mm^3^) were dissected and immersed in 2.5% glutaraldehyde at 4 °C. The liver tissues fixed with glutaraldehyde were rinsed by 0.1M phosphate buffer (pH 7.4) three times and then fixed with 1% osmium tetroxide at 25 °C for 2 h. After rinsing the liver tissue with 0.1M phosphate buffer (pH 7.2) three times, the tissue was dehydrated with gradient ethanol solutions, permeated with acetone/epoxy at 37 °C for 12 h, embedded in epon and sections of 80 nm were cut and stained with uranyl acetate and lead citrate. Finally, images of all liver samples were observed and taken using transmission electron microscope (Tecnai, FEI, Hillsboro, OR, USA).

### 4.6. Immunohistochemistry Staining Assay

Liver tissues were fixed in 4% paraformaldehyde at 25 °C for 48 h, all of which were subsequently embedded in paraffin, sectioned, and stained with anti-CK19, anti-NF-κB p65 antibodies, respectively. After the slices were incubated with the second antibody at 25 °C for 1 h, diaminobenzidine (DAB) staining was then performed. The slices were counterstained with hematoxylin and dehydrated and mounted. Images were obtained using a microscope (ECLIPSE E100, Nikon, Japan).

### 4.7. Cell Line and Cell Culture

Human hepatocyte cell line L02 was obtained from the Cell Bank of Type Culture Collection of the Chinese Academy of Sciences (Shanghai, China). The cells were cultured in Dulbecco’s modified eagle’s medium (DMEM, Gibco, Grand Island, NY, USA) with 10% fetal bovine serum (FBS, Gibco, Grand Island, NY, USA) and 100 U/mL of penicillin (Genom, Hangzhou, China) and 100 μg/mL of streptomycin (Genom, Hangzhou, China) in a 5% CO_2_ and 95% air incubator at 37 °C.

### 4.8. Cell Viability Assay

Cell viability was assessed by using cell counting kit-8 (CCK-8, TargetMol, Boston, MA, USA) according to the manufacturer’s protocols. Briefly, 3 × 10^3^ cells in 100 μL complete medium were seeded in 96-well plates. After being incubated overnight, cells were treated with EE or KD for 24, 48 h, and then CCK-8 solution with 10 μL/well was added to each well and incubated for 1 h at 37 °C. The optical density (O.D) values at 450 nm of each well were determined using a microplate reader. The values of O.D 450 nm were determined to indicate cell viability.

### 4.9. Western Blotting

Total proteins from liver samples or L02 cells were extracted by using RIPA lysis buffer (Beyotime, Shanghai, China), containing protease inhibitors. The proteins in supernatant were obtained by centrifugation at 4 °C for 10 min and stored at −80 °C. The protein concentration was quantified using a BCA protein assay kit (Beyotime, Shanghai, China). Equal amounts of protein samples were subjected to SDS-PAGE and separated according to the molecular weight, which were subsequently transferred to PVDF membranes. After blocking in a TBST buffer, containing 5% nonfat-dried milk powder for 1 h at room temperature, the PVDF membranes were incubated with the primary antibodies overnight at 4 °C and subsequently with the horseradish peroxidase (HRP)-conjugated secondary antibodies for 1 h at room temperature. Protein expressions were developed by an enhanced chemiluminescence (ECL) approach and images of which were visualized using the GeneGnome 5 imaging system (Gene, Hong Kong, China).

### 4.10. Immunofluorescence Staining Assay

Paraffin blocks of liver tissue were cut into 5 μm sections, all of which were subjected to dewaxing, and antigen repair and blocking in 3% BSA. Primary antibody against FXR, relevant secondary antibody and DAPI (4′,6-diamidino-2-phenylindole, Servicebio, China) were used to generate fluorescent staining. All samples were mounted and collected images by a fluorescence microscope (Olympus, Tokyo, Japan).

### 4.11. Statistical Analysis

All data were analyzed by GraphPad Prism 7.0 software (San Diego, CA, USA) and are presented as mean ± SD. Comparisons between the means of groups were assessed by two-tailed Student’s *t*-test or one-way analysis of variance (ANOVA), as stated in the figure legends. The *p* values *<* 0.05 were considered statistically significant.

## 5. Conclusions

Kinsenoside (KD) is a natural small molecule compound that has multiple bioactivities including anti-inflammatory and hepatoprotection. We have demonstrated that KD alleviates 17α-ethinylestradiol-induced cholestatic liver injury in rats, the underlying mechanism of which may be due to the inhibition of inflammatory responses and regulation of FXR-mediated bile acid homeostasis. KD should be investigated as a promising drug candidate for the treatment of cholestasis.

## Data Availability

Data are contained within the article.
